# Role of HER2-Targeted Agents in Adjuvant Treatment for Breast Cancer

**DOI:** 10.1155/2011/730360

**Published:** 2011-09-25

**Authors:** Toru Mukohara

**Affiliations:** Department of Medical Oncology/Hematology, Cancer Center, Kobe University Hospital, 7-5-2, Kusunoki-cho, Chuo-ku, Kobe 650-0017, Japan

## Abstract

Approximately 20% of breast cancers overexpress human epidermal growth factor receptor 2 (HER2) protein, mainly as a result of gene amplification. The receptor tyrosine kinase is believed to play a critical role in the pathogenesis and further proliferation of these tumors. The application of trastuzumab, a humanized monoclonal antibody against the extracellular domain of HER2 protein, to HER2-positive metastatic breast cancer has significantly improved treatment outcomes. Following this success, several phase III trials have evaluated the role of trastuzumab in the adjuvant setting, with the result that trastuzumab use is now the standard of care for most HER2-positive early breast cancer patients. In this paper, we review these pivotal phase III trials. We also discuss unresolved issues in adjuvant treatment with trastuzumab, including target patient population, sequential or concurrent use with chemotherapy or radiation, treatment duration, cardiotoxicity, and the possibility of eliminating chemotherapy. Following confirmation of its ability to partially overcome trastuzumab resistance, we also discuss the role of lapatinib in adjuvant use.

## 1. Introduction

Breast cancer is the leading cause of cancer death among women worldwide, with approximately one million new cases reported each year[1, 2]. Approximately 20% of breast cancer tumors show overexpression of human epidermal growth factor receptor 2 (HER2) protein, which has been repeatedly identified as a poor prognostic factor [3, 4]. Trastuzumab, a humanized monoclonal antibody which targets the extracellular domain of the HER2 protein, has undergone intense clinical investigation since the late 1990s. Clinical development of trastuzumab was initially focused on treatment for HER2-positive metastatic breast cancers in the mid-1990s. In the first pivotal phase III study, patients were treated with doxorubicin or epirubicin and cyclophosphamide (AC or EC) or paclitaxel with (*N* = 235) or without (*N* = 234) trastuzumab [5]. Results for the trastuzumab-containing arm were superior to those with chemotherapy alone in virtually all efficacy parameters assessed, including overall survival (median, 25.1 versus 20.3 months, *P* = 0.046; HR, 0.80) [5], which, together with the results of other studies, led to the broad clinical adoption of trastuzumab in single or combined use with anthracyclines, taxanes, vinorelbine, or capecitabine for HER2-positive metastatic breast cancer [6–8]. Following this success in the metastatic setting, ongoing evaluation of trastuzumab in adjuvant settings for operable HER2-positive breast cancers was started, and with the six randomized phase III trials of the integration of trastuzumab into standard adjuvant treatments completed to date, all but one reporting that the addition of trastuzumab provided a significant improvement in efficacy [9–16].

In this paper, we discuss the current status of trastuzumab therapy in adjuvant treatment for breast cancer. We also discuss unresolved issues in adjuvant use, including target patient population, sequential or concurrent use with chemotherapy, duration of treatment, cardiotoxicity, and the possibility of eliminating chemotherapy. Given that lapatinib, a dual inhibitor of epidermal growth factor receptor (EGFR)/HER2 tyrosine kinase, has been clinically proven to overcome at least some resistance to trastuzumab in the metastatic setting [17], we also discuss the potential role of lapatinib in adjuvant treatment, along with the current status of a large ongoing randomized study.

## 2. Pathophysiology of the HER Family in Breast Cancer

HER2 is a receptor tyrosine kinase (RTK) belonging to the HER family which consists of HER 1 through 4. With the exception of HER2, each member of the HER family has cognate-identified ligands. Ligand binding to extracellular domains induces conformational changes in the receptor, which enable the receptor to form a homo- or heterodimer and to become active as an RTK. Each dimer can subsequently trigger various intracellular signaling pathways, including those of PI3K/Akt, Ras/Raf/MEK/ERK, and STATs, which all play important roles in cellular oncogenic processes such as proliferation, survival, motility, and angiogenesis. 

Because of the lack of a cognate ligand, HER2 must dimerize with other HER family members under physiological conditions. HER2 is considered to be the preferred dimerization partner for the other HER family members. In addition, under conditions of overexpression, HER2 can be constitutively active and transform normal cells in the absence of a ligand [18, 19]. While HER2 can theoretically form four different types of dimers, namely, with HER1, HER2, HER3, or HER4, the HER2/HER3 heterodimer is considered to be the most mitogenic and transforming [20–23].

HER2 is overexpressed in 20%–25% of breast cancers. In preclinical studies, HER2 overexpression has been shown to be associated with increased metastatic potential [24] and resistance to chemotherapeutic drugs such as paclitaxel, docetaxel, doxorubicin, 5-fluorouracil, and etoposide [25, 26]. Clinically, HER2-overexpression has been shown to be associated with poor disease-free survival and overall survival, and poor responsiveness to CMF- (cyclophosphamide, methotrexate, and 5-fluorouracil) like therapy [3, 4, 27, 28]. Gene amplification is considered the main mechanism of HER2 protein overexpression. HER2 has been reported to require HER3 to drive breast cancer cell proliferation, emphasizing the importance of the HER2/HER3 heterodimer complex mentioned above [29].

## 3. Mechanisms of Action of Trastuzumab

The mechanisms of action for trastuzumab can be roughly divided into two categories: inhibition of intracellular signaling and induction of an immune system-mediated antitumor response. Given HER2's effect in triggering multiple signaling pathways, its inhibition should theoretically lead to the inactivation of those pathways. Although it is not fully understood how trastuzumab inhibits HER2 activity, some studies have suggested that the drug might promote internalization and degradation of HER2 [30, 31]. Recent studies have suggested that the HER2/HER3/PI3K complex and subsequent PI3K-Akt signaling pathway play central roles in cell proliferation in HER2-overexpressing cells and that the disruption of this complex might accordingly be the key molecular mechanism of action of trastuzumab [32, 33]. Supporting the involvement of PI3K-Akt pathway in trastuzumab action, studies suggested that activating mutation of the *PIK3CA* gene, which codes the p110*α* catalytic domain of PI3K, or loss of phosphatase and tensin homolog deleted from chromosome 10 (PTEN), could cause resistance to trastuzumab [34–36].

Data from several *in vivo* experiments have indicated that trastuzumab is capable of mediating the induction of immune responses such as antibody-dependent cellular cytotoxicity (ADCC) and complement-dependent cytotoxicity [37]. With ADCC, immunoeffector cells expressing the Fc*γ* receptor recognize and bind to the Fc domain of the IgG1 antibody (trastuzumab) and subsequently lyse cells (in this case, tumor cells) attached to the antibody. Directly proving the role of ADCC in trastuzumab's activity is naturally difficult in patients. However, perhaps the most convincing evidence of the contribution of ADCC to trastuzumab-induced antitumor activity was provided by mice lacking the FC receptor (FcR^−/−^), in which trastuzumab treatment resulted in significantly lower rates of tumor regression than in FC receptor-expressing mice [38]. 

## 4. Role of Trastuzumab in Adjuvant Treatment for Early Breast Cancer

### 4.1. Overview of Pivotal Phase III Randomized Trials

**Figure 1:**
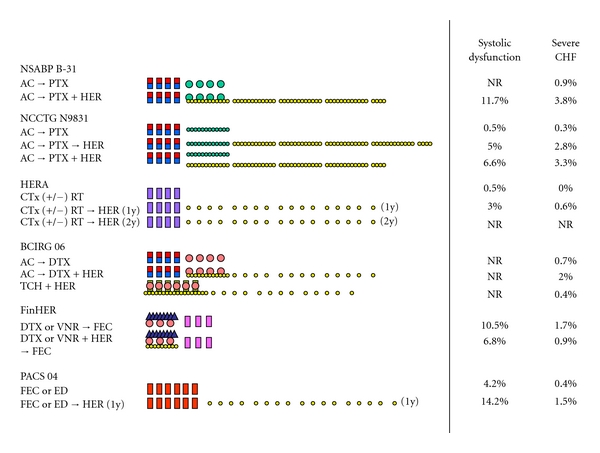
Schema of treatment regimens and associated cardiotoxicity in the large adjuvant trastuzumab trials. AC, doxorubicin 60 mg/m^2^ and cyclophosphamide 600 mg/m^2^, q3w; PTX, paclitaxel 175 mg/m^2^, q3w (NSABP B-31) or 80 mg/m^2^, qw (NCCTG N9831); HER, trastuzumab loading dose of 4 mg/kg followed by 2 mg/kg, qw (NSABP B-31, NCCTG N9831, and FinHER and in combination with DTX in BCIRG 06) or loading dose of 8 mg/kg followed by 6 mg/kg, q3w (HERA and PACS 04 and at trastuzumab alone phase in BCIRG 006); CTx, adjuvant and/or neoadjuvant chemotherapy; RT, adjuvant radiation therapy; DTX, docetaxel 100 mg/m^2^, q3w; TCH, docetaxel 75 mg/m^2^ and carboplatin area under the curve (AUC) 6, q3w and trastuzumab loading dose of 4 mg/kg followed by 2 mg/kg, qw; VNR, vinorelbine, 25 mg/m^2^, qw; FEC, fluorouracil 600 mg/m^2^, epirubicin 60 mg/m^2^, and cyclophosphamide 600 mg/m^2^, q3w (FinHER) or fluorouracil 500 mg/m^2^, epirubicin 100 mg/m^2^, and cyclophosphamide 500 mg/m^2^, q3w (PACS 04); ED, epirubicin 75 mg/m^2^ and docetaxel 75 mg/m^2^, q3w; CHF, congestive heart failure; NR, not reported.

Following its successful application in HER2-positive metastatic breast cancer, trastuzumab was subsequently tested in adjuvant use. As of today, the results of six well-designed phase III trials comparing nontrastuzumab adjuvant therapies with trastuzumab-containing therapies are available in either conference abstract form or full paper (Table 1 and Figure 1).

The National Surgical Adjuvant Breast and Bowel Project trial B-31 (NSABP B-31) compared four cycles of doxorubicin and cyclophosphamide (AC) followed by four cycles of triweekly paclitaxel (tri-PTX) (Arm 1, reference arm) with the same regimen plus 52 weeks of weekly trastuzumab (wkly-HER) beginning on day 1 of tri-PTX (Arm 2) (Figure 1) [10, 13]. Women with estrogen-receptor- (ER) positive or progesterone-receptor- (PgR) positive tumors received 20 mg of tamoxifen per day for five years. Tamoxifen was originally initiated on day 1 of the first cycle of AC, but this was amended in response to the findings of Southwest Oncology Group trial 8814 to require that hormonal therapy be started after chemotherapy [39]. A second amendment following the report of the arimidex, tamoxifen alone or in combination (ATAC) trial permitted treatment with anastrozole in postmenopausal ER- or PgR-positive patients [40]. The North Central Cancer Treatment Group trial N9831 (NCCTG N9831) compared four cycles of AC followed by 12 weeks of weekly paclitaxel (wkly-PTX) (Arm A, reference arm) with the same regimen plus 52 weeks of wkly-HER either following wkly-PTX (Arm B) or beginning on day 1 of wkly-PTX (Arm C) (Figure 1) [10, 41]. Women with ER- or PgR-positive tumors were originally scheduled to receive 20 mg of tamoxifen per day for five years, initiated after chemotherapy. Following the ATAC trial, however, the protocol was amended to permit postmenopausal ER- or PgR-positive patients to be treated with any aromatase inhibitor. Because of the similarity of Arms 1 and 2 in the NSABP B-31, and Arms A and C in the NCCTG N9831, combined analysis was performed and the results were published as a full paper [10], with subsequent updated data available in a conference abstract form [13]. Individual data of the NCCTG N9831 is only available as a conference abstract at this time [41, 42]. 

The Breast Cancer International Research Group 006 (BCIRG 006) compared four cycles of AC followed by four cycles of triweekly docetaxel (tri-DTX) (reference arm) with the same regimen plus 52 weeks of trastuzumab (weekly at DTX phase and triweekly thereafter) beginning on day 1 of tri-DXT (Figure 1). This trial is distinguished from the others by the involvement of a nonanthracycline regimen, consisting of docetaxel, carboplatin, and trastuzumab (TCH), with the aim of reducing cardiotoxicity [11, 12]. The results of this study are presently available only as a conference abstract [11, 12].

The Herceptin Adjuvant (HERA) study differed from the other trials in that patients were randomized at the point of completion of standard adjuvant chemotherapy with or without radiotherapy to either observation alone, one year of triweekly trastuzumab (tri-HER), or two years of tri-HER (Figure 1) [9, 14]. Adjuvant endocrine therapy, primarily 20 mg of tamoxifen per day, was given after chemotherapy to women with ER- or PgR-positive disease. An amendment to the protocol allowed aromatase inhibitors to be used instead of or in sequence with tamoxifen [9]. Initial and two follow-up data from observation and one year of trastuzumab were published in 2005, 2007, and 2011, respectively [9, 14, 43]. Data from two years of trastuzumab is not available at this point. 

The Finland Herceptin (FinHER) study was designed to compare three cycles of triweekly vinorelbine with tri-DXT, both followed by three cycles of fluorouracil, epirubicin, and cyclophosphamide (FEC) (Figure 1) [15, 16]. For HER2-positive patients, patients were additionally randomized at the tri-VNR or tri-DXT phase to receive or not receive tri-HER. Patients with ER- or PgR-positive tumors initially received 20 mg of tamoxifen per day for five years. The protocol was amended during the trial to allow the switching of tamoxifen to an aromatase inhibitor for postmenopausal women after 2 to 3 years of tamoxifen use to complete the 5-year administration of a hormonal agent and to allow administration of an aromatase inhibitor for a further 2 to 3 years after the completion of 5-year administration of tamoxifen [15]. Distinct from the other trials, trastuzumab was given for only nine weeks in this study. Initial results were published in 2006 and the final results in 2009 [15, 16].

The Programmes d'Actions Concertées Sein (PACS) 04 trial first randomly assigned patients to six courses of fluorouracil, epirubicin 100 mg/m^2^, and cyclophosphamide (FEC100) or six courses of epirubicin 75 mg/m^2^ and docetaxel (ED75) and then further randomized HER2-positive patients to sequential tri-HER for 1 year or to observation (Figure 1) [44]. Endocrine therapy was mandatory for patients with ER- or PgR-positive tumors. Premenopausal patients were given 20 mg of tamoxifen per day for 5 years, while postmenopausal patients received endocrine therapy with either anastrozole or tamoxifen at the discretion of investigator [44].

### 4.2. Efficacy Data

Although the PACS 04 trial enrolled only node-positive patients, other baseline characteristics of the disease were quite similar between the six trials (Table 1). All six trials established recurrence-free survival (RFS) (FinHER) or disease-free survival (DFS) (the others) as the primary endpoint with subtle difference in the definition regarding whether to count ductal carcinoma in situ (DCIS) or second nonbreast malignancy as an event [9, 10, 16]. Although in the final report of the FinHER study the primary endpoint was amended to be distant disease-free survival [15], we adopted the efficacy data from the initial report in Table 1 for better comparison between the trials. 

All but the PACS 04 study showed the superiority of trastuzumab-containing regimens over standard regimens without trastuzumab, with statistical significance, with the most recent hazard ratios (HRs) ranging from 0.42 to 0.76 (Table 1) [12–14, 16, 41, 44]. In PACS 04, trastuzumab did not significantly improve DFS with HR of 0.86 (95% CI 0.61–1.27; *P* = 0.41) [44]. Overall survival (OS), set as a secondary endpoint in each trial, tended to be improved with trastuzumab-containing regimens compared to nontrastuzumab regimens, again with the exception of the PACS 04 trial (Table 1). The most recent HRs for OS in trials other than PACS 04 ranged from 0.41 to 0.86, and *P* values were less than 0.05, except for those for Arm B (sequential trastuzumab) in the NCCTG N9831, and trastuzumab-containing regimens employed in the HERA and FinHER studies (Table 1) [12–14, 16, 41, 44]. HR for OS in PACS 04 was 1.27 with the wide 95% CI of 0.68–2.38, possibly due to small sample size (Table 1).

### 4.3. Target Patients for Treatment with Trastuzumab 

#### 4.3.1. HER2 Measurement

With the clinical development of anti-HER2 agents, including trastuzumab, methods for evaluating the expression level of HER2 has also evolved. In the six trastuzumab adjuvant trials, HER2 positivity was evaluated with immunohistochemistry (IHC), fluorescence in situ hybridization (FISH), or chromogenic in situ hybridization (CISH), either alone or on combination. All six trials stipulated that central confirmation of HER2-positivity using these methods was mandatory either from the beginning or after protocol amendment [44, 45].

IHC is a semiquantitative method which detects HER2 protein levels on the cell surface in breast cancer specimens. Currently, the US Food and Drug Administration (FDA) has approved three types of IHC system to determine HER2 status: HercepTest (Dako, Glostrup, Denmark), Pathway anti-HER2/neu (Clone CB11) (Ventana-Roche, Tucson, Ariz, USA), and InSite HER2/neu CB11 (Biogenex, San Ramon, Calif, USA). In each system, IHC scoring is performed subjectively as 0, 1+, 2+, or 3+ based on the intensity of membrane staining in >10% of cancer cells [45]. 

FISH or CISH is a DNA-based assay that directly measures HER2 gene amplification. FISH testing results are semiquantitative, based on the absolute number of HER2 signals or the average ratio of HER2 signals to CEP17 (a probe against the chromosome 17 centromeric sequences) signals in nonoverlapping interphase nuclei of the lesion. The FDA has approved three types of FISH testing: PathVysion (Vysis, Downers Grove, Ill, USA), in which a ≥2.0 ratio HER2/CEP17 is considered amplified; INFORM (Ventana-Roche), in which ≥5.0 gene copies of HER2 is considered amplified; and HER2 FISH pharmDx (Dako), in which a ≥2.0 ratio of HER2/CEN-17 is considered amplified. In addition, one CISH test has been approved: SPOT-Light (Invitrogen, Carlsbad, Calif, USA), in which 10 dots, or large clusters, or a mixture of multiple dots and large clusters in 50% of tumor cells is considered amplified.

To improve the accuracy of HER2 testing and its utility as a predictive marker for anti-HER2 agents, the American Society of Clinical Oncology/College of American Pathologists (ASCO/CAP) updated their guideline recommendation for HER2 testing in 2007 [45]. Positivity for HER2 is demonstrated by either IHC HER2 3+, defined as uniform intense membrane staining of >30% of invasive tumor cells or FISH amplified, defined as a ratio of HER2 to CEP17 >2.2, or an average HER2 gene copy number >6 signals/nucleus for those test systems without an internal control probe. They also recommend that if initial results for HER2 are equivocal, that is, HER2 2+, IHC or FISH ratio 1.8–2.2, or HER2 gene copy 4.0–6.0, validated IHC, FISH, or CISH should be performed or repeated. 

It should be noted that the new ASCO/CAP HER2 positivity criteria are stricter than those of the manufacturers. In the five adjuvant trastuzumab trials with the exception of PACS 04, positive IHC membrane staining in 10% to less than 30% of cells and an HER2/CEP17 ratio of 2–2.2, were eligible based on the manufacturers' criteria. In the PACS 04 trial, while the same manufacturers' criteria were used for IHC, only IHC 2+ cases were sent to FISH and HER2/CEP17 ratio ≥2.2 was defined as positive [44]. But, these cases would have been equivocal based on the ASCO/CAP guideline. To assess the influence of differential criteria on study result, tumor samples in the NCCTG N9831 were reevaluated using the ASCO/CAP criteria [42], but the results showed that only a small percentage (by IHC, 3.7%; FISH, 1.4%; both, 1.7%) of patients did not meet the ASCO/CAP 2007 HER2 positivity guidelines [42]. Because there is no evidence to support that the patients with equivocal HER2 positivity should be excluded from adjuvant treatment with trastuzumab, the drug is generally given as a part of systemic therapy.

#### 4.3.2. Definition of High Risk no Disease

Among the six trials, only the PACS 04 trial enrolled only node-positive patients, whereas the other five trials enrolled both node-positive and negative high-risk patients. However, they varied in their definition of high-risk. Both the NCCTG N9831 and NSABP B-31 trials defined tumors of more than 2 cm in diameter for hormone receptor- (HR-) positive disease and more than 1 cm in diameter for HR-negative disease as high risk (Table 1). The HERA study enrolled node-negative patients with primary tumors that were more than 1 cm regardless of HR status, while the BCIRG 006 trial considered high risk as node-negative disease with tumors that were either more than 2 cm in diameter, HR-negative, or histological grade (HG) 2 or 3, or with a patient age of younger than 35 years. Finally, the FinHER study enrolled node-negative patients with tumors that were more than 2 cm in diameter and PgR-negative. Of note, no trials involved node-negative disease with a primary tumor size of 1 cm or less, and no definitive threshold for treatment with or without trastuzumab has accordingly yet been established.

The often-utilized St Gallen International Expert Consensus, renewed in 2009, included the following statement: “*patients with tumors of <1 cm in size without axillary nodal involvement and without other features indicating increased metastatic potential (e.g. vascular invasion) might not need adjuvant systemic therapy” *[46]. The National Comprehensive Cancer Network (NCCN) Clinical Practice Guideline, on the other hand, recently raised its category of evidence and consensus from Category 3A (reflecting the presence of major disagreement among NCCN panel members) to Category 2A (based on a lower-evidence but uniform NCCN consensus) for consideration of adjuvant trastuzumab in women with node-negative tumors that are 0.6 to 1.0 cm (NCCN, Version 2, 2011). This update was based on several retrospective studies which suggested that HER2-positivity is a poor prognostic factor even in patients with node-negative tumors ≤1.0 cm [47–49]. The consideration of trastuzumab use for node-negative small tumors is also supported by a recently published article by Banerjee et al. [50]. These authors reviewed retrospective studies which followed the outcome of patients with HER2-positive, node-negative, and 1 cm or smaller tumors [50]. They found that while relapse in patients with HER2-positive small tumors was less than 10% at 10 years after diagnosis, it rose to 16% to 17% and 21% to 29% at 15 and 20 years after diagnosis, respectively, in those studies with long-term followup [50]. They also noted that patients with relatively small tumors (1.1–2.2 cm) in the HERA study had the same magnitude of risk reduction with trastuzumab [50]. Therefore, although a final decision on the used of trastuzumab in these patients should be left to clinical judgment, physicians should at least discuss this issue with patients.

### 4.4. Treatment Duration

In addition to one-year trastuzumab and reference arms, the HERA study also established a 2-year trastuzumab arm [9]. No efficacy or safety data for this longer arm is yet available, so the value of administration beyond one year is unclear. The FinHER study was distinguished from the other large trials by its administration of trastuzumab for only 9 weeks, concurrently with vinorelbine or docetaxel [15, 16]. Despite this short duration, trastuzumab provided a dramatic improvement in efficacy over the nontrastuzumab control, with an HR of 0.42 for RFS [16]. Multiple randomized trials comparing the standard 1 year of trastuzumab therapy with a shorter duration are ongoing. In one, the synergism or long duration (SOLD) Study conducted by the Finnish group following the FinHER trial, three cycles of tri-DXT in combination with wkly- or tri-HER followed by three cycles of FEC was compared with the same regimen followed by 14 doses of additional tri-HER to give a total duration of trastuzumab therapy of one year. Results of these trials will provide an insight into the optimal duration of adjuvant trastuzumab (NCT00593697). Until then, however, one-year trastuzumab should be considered the current standard, because this was the duration used by the majority of the randomized trials.

### 4.5. Concurrent or Sequential

Joint analysis of the NCCTG N9831 and NSABP B31 trials, which compared a nontrastuzumab control and trastuzumab given concurrently with paclitaxel, produced the impressive HR for DFS of 0.49 [13]. The NCCTG N9831 involved another arm in which trastuzumab was given sequentially after the completion of wkly-PTX (Arm B, Figure 1). Individual data for this trial comparing reference Arm A (nontrastuzumab) with sequential arm (Arm B) is available in conference abstract form, and show an HR for Arm B of 0.67, which is somewhat less robust than that in the joint analysis of NCCTG N9831 and NSABP B31 trials [41]. Further, the concurrent arm was superior to the sequential arm, with an HR for DFS of 0.75 (95% CI 0.60–0.94; *P* = 0.0190) [41] even though this comparison was not the primary analysis of the NCCTG N9831. In addition, the HERA study, in which trastuzumab was given sequentially after completion of standard chemotherapy, again produced the less robust HR for DFS of 0.76, which is less than the joint analysis of the NCCTG N9831 and NSABP B31 trials. Further, in PACS 04 trial, trastuzumab given after completion of six courses of FEC100 or ED did not provide any significant impact on DFS compared to observation. Therefore, it is very likely that trastuzumab is more active when given concurrently with than sequentially after chemotherapy as long as the same chemotherapy regimen is employed.

### 4.6. Cardiotoxicity

Although trastuzumab is generally very well tolerated, it occasionally impairs cardiac function due to myocardium damage. While anthracycline-induced cardiotoxicity is dose-dependent and results mainly from oxidative mechanisms that lead to the apoptosis and necrosis of cardiomyocytes, trastuzumab-induced cardiotoxicity is generally not dose-dependent and does not show the ultrastructural changes typical of anthracycline [51, 52]. Although the mechanism of trastuzumab-associated cardiac dysfunction is not precisely known, it has been suggested that HER2 may have a protective role for cardiomyocytes [53]. No consistent risk factors for trastuzumab-associated cardiotoxicity have been identified, out older age and need for antihypertensive agents baseline left ventricular ejection fraction (LVEF) <55% were found to be associated with incidence of cardiac events in the NCCTG N9831 and NSABP B31 trials [54, 55]. 

As summarized in Figure 1, the incidence of cardiac dysfunction and severe chronic heart failure (CHF) in trastuzumab-containing treatment in the phase III trials ranged from 3.0% to 14.2% and from 0.4% to 3.8%, respectively. Given their differing definitions of cardiac dysfunction and severe CHF, direct comparison between regimens requires prudence. Nevertheless, when we see much lower incidence of cardiotoxicity in the HERA study than that in the NSABP B-31 study, it appears that a high incidence of cardiotoxicity should be expected when trastuzumab is given concurrently with chemotherapy (Figure 1). On the other hand, the direct comparison of concurrent versus sequential administration of trastuzumab in the NCCTG N9831 showed only minimally reduced cardiotoxicity in the latter [55]. The PACS 04 trial also had high incidence of systolic dysfunction despite sequential administration of trastuzumab after completion of six cycles of FEC100 or ED75 [44]. Considering the fact that in the HERA study only one-fourth of patients took taxanes before trastuzumab and median cumulative dose of anthracyclines were relatively low (238 and 405 mg/m^2^ for doxorubicin and epirubicin, respectively), high dose of anthracycline and a taxane before initiating sequential trastuzumab may be associated with higher cardiotoxicity. Relevant to this, trastuzumab was given before anthracycline-based chemotherapy in the FinHER study only, which had a very low incidence of severe CHF (one patient) [15]. Although this may have been caused by the shorter duration of trastuzumab therapy than in the other trials, as discussed below, this, nevertheless, supports the possibility that trastuzumab after anthracycline therapy hampers the heart's repair mechanisms.

In the BCIRG 006 trial, a nonanthracycline regimen (TCH) was included owing to concerns of cardiotoxicity caused by trastuzumab use following anthracycline. This regimen was indeed associated with lower cardiotoxicity than those containing anthracycline [11, 12]. It should be noted, however, that decrease in risk with TCH appears somewhat smaller than that with AC followed by docetaxel/trastuzumab (HR for DFS, AC followed by docetaxel/trastuzumab versus TCH, 0.64 versus 0.75), notwithstanding that the trial was not designed to compare these two regimens [12]. 

Another potential approach to avoiding trastuzumab-associated cardiotoxicity is to shorten the duration of therapy, as attempted in the FinHER study. These investigators appear to have achieved this particular goal, with even a lower incidence of systolic dysfunction and symptomatic CHF in the HER2-containing arm than in the nontrastuzumab control arm [16]. As discussed above, however, the degree to which duration can be shortened without decrementing efficacy remains unknown.

Radiation after breast surgery has been considered a cardiac risk. While in the HERA and PACS 04 studies and the FinHER study postsurgical radiotherapy was given sequentially before and after trastuzumab, respectively, it was done concurrently with trastuzumab in the other four trials. As the HERA and FinHER studies had a lower incidence of cardiotoxicity, it remains possible that the concurrent use of radiotherapy and trastuzumab is cardiotoxic. On the other hand, however, an unplanned analysis of patients treated in the N9831 trial revealed no difference in the frequency of cardiac events between patients treated with or without radiotherapy even in the trastuzumab-containing arm [56]. The delivery of radiation therapy concomitantly with trastuzumab is therefore generally considered safe.

### 4.7. Adjuvant Trastuzumab without Chemotherapy

Consideration has been given to the possibility of adjuvant trastuzumab therapy without chemotherapy, particularly when the patient is old or has a comorbidity, or the tumors are node-negative, ER- or PgR-positive, and small. In the metastatic setting, a phase III trial comparing anastrozole plus trastuzumab with anastrozole alone as first-line therapy for postmenopausal HER2- and hormone receptor-positive breast cancer showed combination therapy was superior in terms of progression-free survival (PFS) and response rate [57]. However, the combination of endocrine therapy and trastuzumab without chemotherapy remains unproven in the adjuvant setting, and further data is accordingly needed before the elimination of chemotherapy can be justified. In this regard, a phase III trial comparing trastuzumab monotherapy with trastuzumab plus chemotherapy in HER2-positive elderly breast cancer patients is currently underway (NCT01104935).

## 5. Role of Lapatinib in Adjuvant Settings

### 5.1. Rationale of Lapatinib Use in Adjuvant Therapy for HER2-Positive Breast Cancer

Lapatinib is a dual EGFR/HER2 tyrosine kinase inhibitor [58]. In preclinical models, lapatinib has been shown to overcome resistance to trastuzumab, inhibiting phosphorylation of HER2 and overall growth in HER2-overexpressing breast cancer cell lines specifically selected for their *in vitro* resistance to trastuzumab [59]. Additionally, lapatinib inhibited growth of cells that express p95 HER2, which is truncated and lacks an extracellular domain and, therefore, has the potential to develop resistance to trastuzumab [60, 61]. Supporting the role of lapatinib as a backup drug for trastuzumab, a randomized phase III trial comparing capecitabine in combination with lapatinib to capecitabine alone in 399 patients with HER2-positive recurrent/metastatic breast cancer who had progressed with the anthracycline, taxane, and trastuzumab, reported that overall response was superior in the lapatinib-receiving arm (23.7% versus 13.9%; *P* = 0.017), as was median time to progression (27.1 versus 18.6 weeks, *P* < 0.001), while overall survival tended to be longer [62].

Unlike trastuzumab, lapatinib has been suggested to cross the blood-brain barrier, which rationalizes its use in patients with central nervous system (CNS) metastases. In a phase II trial of lapatinib in 39 patients with HER2-positive breast cancer with brain metastases, two patients experienced partial response based on the response evaluation criteria in solid tumors (RECISTs) criteria, and five additional patients experienced at least a 30% shrinkage of CNS lesions [63].

With regard to cardiac safety, prospective data collected in 44 clinical studies of lapatinib (*N* = 3,689) revealed a low incidence of cardiac events potentially caused by lapatinib (1.6%) [64].

### 5.2. Rationale of Lapatinib in Combination with Trastuzumab

A preclinical study of lapatinib in combination with trastuzumab reported that survivin was more strongly downregulated and apoptosis was more strongly induced with this combination than with either agent alone [65]. Another preclinical study showed that lapatinib alone or in combination with trastuzumab trastuzumab inhibited HER2 phosphorylation, prevented receptor ubiquitination, and resulted in the accumulation of inactive HER2 at the cell surface of HER2-overexpressing breast cancer cell lines, which led to the subsequent enhancement of ADCC by trastuzumab [66]. Consistent with the preclinical data, a recent phase III clinical trial comparing lapatinib and trastuzumab in combination to lapatinib alone in HER2-positive metastatic breast cancer patients who had progressed on trastuzumab-based regimens reported that progression-free survival as the primary endpoint was better in the combination arm than in that with lapatinib alone (hazard ratio [HR] = 0.73; 95% CI, 0.57 to 0.93; *P* = .008) [67]. Furthermore, in the neoadjuvant lapatinib and/or trastuzumab treatment optimisation (Neo-ALTTO trial), 455 preoperative breast cancer patients with HER2-positive tumors received an initial 6 weeks of lapatinib, trastuzumab, or their combination, followed by another 12 weeks of the same anti-HER2 therapy plus wkly-PTX prior to definitive surgery. The first results, presented at the San Antonio Breast Cancer Symposium (SABCS) 2010, showed the highest pathological complete response (pCR) rate, the primary endpoint of the trial, in the combination arm (pCR rate [%]; lapatinib versus trastuzumab versus combination; 24.7 versus 29.5 versus 51.3; *P* = .34 for lapatinib versus trastuzumab and *P* = .001 for trastuzumab versus combination).

While this accumulating evidence supports the superiority of lapatinib and trastuzumab combination to either therapy alone, its role in adjuvant therapy remains to be revealed in the ALTTO study, as discussed below.

### 5.3. ALTTO Trial

**Figure 2:**
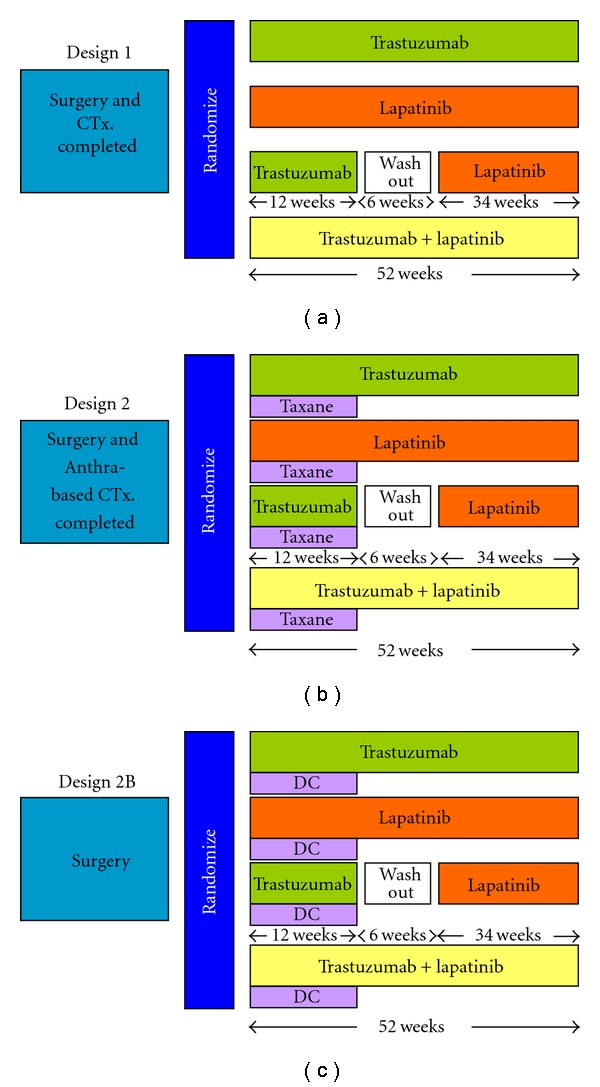
Schema of the ALTTO trial; DC, docetaxel and carboplatin.

The ALTTO trial is a large, randomized phase III trial designed to evaluate the role of lapatinib in adjuvant treatment for HER2-positive breast cancer patients (Figure 2) [68]. Node-positive and node-negative patients with tumors ≥1.0 cm in greatest diameter were eligible, similar to the trials of adjuvant trastuzumab. Unlike these trials; however, HER2-positivity was evaluated following the ASCO/CAP guideline, so that patients with equivocal HER2-positivity were excluded. The ALTTO trial is comparing trastuzumab for 1 year as the reference arm with either lapatinib for 1 year, trastuzumab for 6 months followed by lapatinib for 6 months, and lapatinib in combination with trastuzumab for 1 year. This trial has two pragmatic designs; lapatinib and/or trastuzumab are given after the completion of anthracycline-based chemotherapy (Design 1, Figure 2), concomitantly with taxanes (Design 2, Figure 2), or with a nonanthracycline regimen (docetaxel plus carboplatin) (Design 2B, Figure 2). Designs 1, 2, and 2B were set referring to the HERA, NSABP B21 and NCCTG N9831, and BCIRG 006 trials, respectively. Designs 1 and 2 have already completed accrual, and Design 2B will complete accrual by early 2011. The results will provide answers about the role of lapatinib in adjuvant treatment for HER2-positive breast cancer. 

## 6. Future Directions

### 6.1. Anti-HER2 Agents in HER2-Negative Population

In early clinical trials of trastuzumab for metastatic breast cancer, patients with HER2 2+ by IHC were enrolled together with HER2 3+ patients. Because subset analysis showed that the benefit of trastuzumab was none or smaller in HER2 2+ patients [5, 69], virtually all subsequent clinical trials of trastuzumab were limited to patients with HER2 3+ or FISH-positive disease. 

In the NSABP B-31 trial, HER2 positivity was initially evaluated based on institutional testing, but excessive false positive HER2 results led to a change to mandatory confirmation by the central office [70]. Among the 1787 patients with follow-up data in the trial, 174 patients had breast cancers that turned out to be HER2-negative (9.7%) on central review [71]. Interestingly, these patients also appeared to benefit from trastuzumab (HR for disease-free survival, 0.34; 95% CI, 0.14 to 0.80; *P* = .014) [71]. Although this analysis was exploratory in a small subset, it at least raised a question of whether the benefit of adjuvant trastuzumab is really limited to patients with HER2-positivity based on today's criteria.

Answering this question will require a phase III trial of adjuvant trastuzumab in women with breast cancers that do not meet established criteria. Additionally, refinement of HER2 testing methodology may be required. In their comparative analysis of 568 breast cancer tumor samples for HER2 with FISH according to the ASCO/CAP guideline and quantitative reverse transcriptase PCR (qRT-PCR) in central laboratories [72], Baehner et al found a high concordance rate of 97% (95% CI, 96%–99%) between the two methodologies [72]. Given that qRT-PCR is faster than FISH and generally more quantitative than FISH or IHC, it might be a candidate for next-generation HER2 testing. 

### 6.2. Newer Anti-HER2 Agents 

#### 6.2.1. Pertuzumab

Pertuzumab is another monoclonal antibody against the extracellular domain of HER2 protein, but it attaches to a different epitope of HER2 from trastuzumab. Pertuzumab is believed to inhibit heterodimer formation between HER2 and EGFR or HER3 [73]. While the HER2/HER3 heterodimer is considered important in HER2-driven cell signaling, a preclinical study showed that the heregulin-dependent HER2/HER3 heterodimer is not disrupted by trastuzumab, but it is disrupted by pertuzumab [32]. In a phase II clinical trial involving combination treatment with pertuzumab and trastuzumab in HER2-positive metastatic breast cancer patients, treatment produced a response rate and disease control rate of 24.2% and 50%, respectively [74]. In the neoadjuvant setting, the neoadjuvant study of pertuzumab and herceptin in an early regimen evaluation (NeoSphere) trial randomized 417 patients with centrally confirmed HER2-positive breast cancer to either docetaxel plus trastuzumab (HD, reference arm, *n* = 107), docetaxel, trastuzumab, and pertuzumab (HDP, *n* = 107), pertuzumab plus trastuzumab (HP, *n* = 107), or docetaxel plus pertuzumab (DP, *n* = 96). Patients received four cycles of therapy prior to surgery. The results presented at SABCS 2010 showed that the pCR rate, the primary endpoint, was significantly higher in the HDP arm and lower in the HP arm compared to the reference HD arm (pCR rate [%]; HD versus HDP versus HP versus DP; 29.0 versus 45.8 versus 16.8 versus 24.0; *P* = .0141 for HD versus HDP). These studies suggest that the addition of pertuzumab to standard trastuzumab-based treatment might enhance efficacy in both metastatic and operable HER2-positive breast cancer. 

#### 6.2.2. Trastuzumab-DM1

Trastuzumab-DM1 is comprised of trastuzumab, DM1, an inhibitor of tubulin polymerization derived from maytansine, and the stable [N-maleimidomethyl]cyclohexane-1-carboxylate (MCC) linker that conjugates DM1 and trastuzumab. The compound is designed to efficiently deliver DM1 to HER2-overexpressing cancer cells. Preclinical studies have demonstrated the growth-inhibitory effect of trastuzumab-DM1 in HER2-overexpressing and trastuzumab-resistant cells [75]. In a phase II trial involving HER2-positive metastatic breast cancer patients who had experienced disease progression despite trastuzumab-based therapy (*n* = 112), trastuzumab-DM1 yielded a response rate and progression-free survival of 26.9% and 4.6 months, respectively [76]. Importantly, trastuzumab-DM1 demonstrated similar antitumor activity and a response rate of 24.2% even in patients previously treated with lapatinib and trastuzumab (*n* = 66) [76]. Feasibility and efficacy of trastuzumab-DM1 in adjuvant or neoadjuvant treatment for HER2-positive early breast cancer are currently under evaluation in a phase II study (NCT01196052). 

#### 6.2.3. HER2 Vaccines

Vaccines and adoptive immunotherapy targeting the HER2 extracellular domain have been tested in clinical trials, with results showing that significant levels of durable T-cell HER2 immunity can be generated with active immunization without significant autoimmunity consequences against normal tissues [77]. Preliminary data from clinical trials testing the potential use of HER2 vaccines in adjuvant therapy for high-risk breast cancer patients have shown promising results [78]. 

## 7. Conclusions

The integration of trastuzumab into conventional adjuvant chemotherapy has significantly improved treatment outcomes in patients with HER2-positive early breast cancer. Although it is impossible to specify one particular regimen as superior, these trials suggest that anthracyclines followed by trastuzumab given concurrently with taxanes appear most promising albeit at the price of a relatively high incidence of cardiotoxicity. With the evidence available today, it is left to clinical judgment to determine whether patients with equivocal HER2-positivity based on ASCO/CAP or with a tumor size of 0.6 to 1.0 cm should be given trastuzumab, and what level of cardiac risk warrants less cardiotoxic trastuzumab regimens, namely, those with a shorter duration of trastuzumab, sequential use, and the avoidance of anthracyclines. The ALTTO trial will clarify the role of lapatinib in adjuvant treatment for HER2-positive breast cancer, as it is or in combination with trastuzumab. Role of newer class of anti-HER2 agent in adjuvant treatment for breast cancer will also be evaluated in clinic in the near future.

## Figures and Tables

**Table 1:** Adjuvant trastuzumab trials.

Trials	*N*	Eligible patient	Pathological tumor size in enrolled patients	pN (UICC) in enrolled patient	ER (+) in enrolled patients	HG in enrolled patients	Median follow-up, years	DFS HR (95% CI, *P* value)	OS HR (95% CI, *P* value)	Reference
NCCTG N9831 and NSABP B-31Joint	3,969		≤20 mm 39% 21–40 mm 45% >40 mm 15%	pN0 53% pN1 27% pN3 14%	52%	HG1, 2% HG2 28% HG3 69%	2.9	0.49 (0.41–0.58, *P* < 0.0001)	0.63 (0.49–0.81, *P* = 0.0004)	[13]
NCCTG N9831 (control versus sequential HER)	2,184	*n* (+) or *n* (−) and *t* > 1 cm for HR (−) and *t* > 2 cm for HR (+)	NR	NR	NR	NR	5.5	0.67 (0.55–0.82, *P* = 0.0005)	0.86 (0.65–1.13, *P* = 0.281)	[41]
NCCTG N9831 (concurrent HER versus sequential HER)							5.3	0.75 (0.60–0.94, *P* = 0.0190)	0.79 (0.59–1.08, *P* = 0.135)	[41]

HERA	5,081	*n *(+) or *n *(−) and *t* > 1 cm	≤20 mm 40% 21–50 mm 44% >50 mm 5%	pN0 32% pN1 29% pN2 28%	46%	HG1 3% HG2 32% HG3 60%	2	0.76 (0.66–0.87, *P* < 0.0001)	0.85 (0.70–1.04, *P* = 0.11)	[43]

BCIRG 006 (control versus DXT+HER)	3,222	*n *(+) or *n *(−) and either *t* > 2 cm, HR (−), HG 2-3, or <35y.o	≤20 mm *≈*40% 21–50 mm *≈*54% >50 mm *≈*6%	pN0 *≈*29% pN1 *≈*39% pN3 *≈*33%	54% (ER and/or PgR)	NR	5.4	0.64 (0.53–0.78, *P* < 0.001)	0.63 (0.48–0.81, *P* = 0.004)	[12]
BCIRG 006 (control versus TCH)								0.75 (0.54–0.90, *P* = 0.04)	0.77 (0.60–0.99, *P* = 0.017)	[12]

FinHER	232	*n *(+) or *n *(−) and *t* ≥ 2 cm and PgR (−)	≤10 mm 7% 1.1–20 mm 28% >20 mm 65%	pN0 16% pN1 53% pN3 31%	47%	HG1 2% HG2 31% HG3 65%	3	0.42 (0.21–0.83, *P* = 0.01)	0.41 (0.16–1.08, *P* = 0.07)	[16]

PACS 04	528	*n *(+)	≤20 mm 44% >20 mm 54%	pN0 0% pN1 58% pN3 42%	60%	HG1 3% HG2 31% HG3 65%	3.9	0.86 (0.61–1.22, *P* = 0.41)	1.27 (0.68–2.38, NR)	[44]

HR: hazard ratio; *n* (+): nodepositive; *n* (−): nodenegative; NR: not reported; UICC: International Union Against Cancer.
